# Nanotoxicology: Emerging Challenges and Future Solutions for Safe Nanomaterial Applications

**DOI:** 10.3390/nano16161020

**Published:** 2026-08-18

**Authors:** Mohamed El Amine Boudjouraf, Anna Sierosławska

**Affiliations:** 1 Independent Researcher; bougou.rakon05@gmail.com; 2 Department of Physiology and Toxicology, Faculty of Medicine, The John Paul II Catholic University of Lublin, Konstantynów Str. 1 I, 20-708 Lublin, Poland

**Keywords:** nanotoxicology, predictive toxicology, safer-by-design, engineered nanomaterials, Quantitative Structure-Activity Relationship (QSAR), risk assessment, in silico modeling

## Abstract

The emergence of nanotechnology has led to a rapid increase in intentional and unintentional exposure to engineered nanoparticles (NPs), raising significant concerns over their impact on humans, animals, and ecosystems. Nanotoxicology has evolved to assess these adverse effects, but the field faces key challenges including complex physicochemical characterization, difficulties in exposure assessment, dynamic biological interactions, and distinct regulatory gaps. To address these challenges, solutions such as the standardization of testing protocols, the adoption of advanced 3D in vitro and in silico modeling, and the implementation of “safer-by-design” principles are proposed. The development of biodegradable nanomaterials (NMs) and effective risk management further emphasizes that responsible development and interdisciplinary collaboration are essential to balance technological innovation with human and environmental safety.

## 1. Introduction

Over the past few decades, nanotechnology has evolved from an emerging science into a cornerstone of modern medicine, accelerating the development and industrial-scale production of engineered nanoparticles. The worldwide economic impact of this transition is substantial; recent market analyses indicate that the global nanomaterials market was valued at over USD 13 billion in 2024 and is projected to exceed USD 33 billion by 2031, heavily driven by the electronics, aerospace, and chemical manufacturing sectors [1]. Furthermore, the specialized nanomedicine sector commands a massive economic footprint valued at over USD 190 billion in 2023, fueled by the escalating clinical demand for advanced healthcare solutions [2].

Because of their incredibly small size and highly tunable physical and chemical properties, nanoparticles are widely utilized as nanomedicines and drug carriers. Engineered from a diverse spectrum of precursors including metals, ceramics, and polymers, these nanoscale entities exhibit unique physicochemical behaviors that fundamentally deviate from their bulk counterparts [3]. This has led to an explosion of nanoparticle applications across various industry sectors and other fields, especially in medicine, where modified nanocarriers have revolutionized targeted drug delivery, medical imaging, and disease diagnostics [4]. However, this rapid, exponential proliferation of new nanomaterials brings a hidden challenge as the properties that make nanoparticles so effective also allow them to interact with biological systems in unpredictable and potentially dangerous ways [5]. For example, the ability of nanoparticles to easily enter the human body and cross protective shields, such as the blood–brain and air–blood barriers, allowing them to invade blood circulation and induce severe toxicological damage to organs like the brain and the heart [3,5]. After penetrating cells, nanoparticles are able to trigger a cascade of structural, metabolic, and genetic disturbances. Extensive toxicological studies show that depending on their size, shape, surface charge, and composition, nanoparticles can severely disrupt the respiratory, nervous, immune and endocrine systems [3,4]. Despite these known risks, the pace of nanoparticle innovation far exceeds our ability to properly test and regulate them [6]. Currently, the primary approach to evaluating nanoparticle safety depends almost entirely on standard in vitro cell assays and in vivo animal testing [3]. To bridge this critical gap, there is an urgent need to modernize how we evaluate and manage nanotoxicity. The objective of this review is to critically examine the mechanisms behind nanoparticle toxicity and advocate for the adoption of highly specific, modernized evaluation frameworks by moving beyond traditional testing; the field must embrace advanced, high-throughput physical models like microfluidic chips, organoids and powerful computational machine learning models that can rapidly predict a particle’s toxicity based on its physical traits [3,7,8]. Ultimately, standardizing these advanced testing models will help implement “safer-by-design” strategies such as surface modification and PEGylation, ensuring that the next generation of nanotechnology is both highly effective and inherently safe for human health [4,9].

## 2. Types and Sources of Nanomaterials

Nanomaterials are broadly classified based on their origin into three main categories: natural, incidental and engineered [6,10].

### 2.1. Natural Nanoparticles (NONPs)

**Figure 1:**
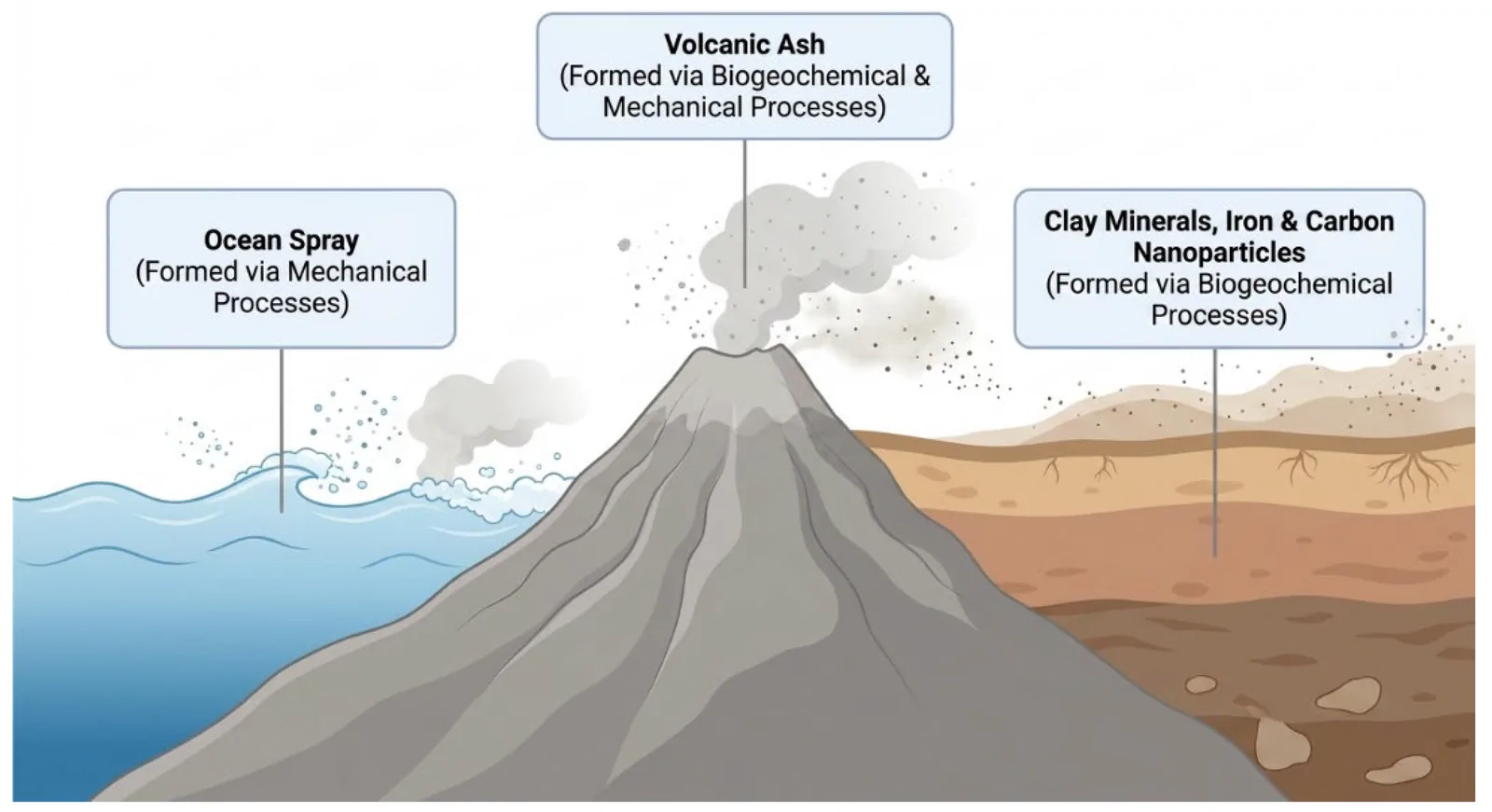
Environmental origins and formation mechanisms of natural nanoparticles [10,11,12].

These nanoparticles exist naturally in the environment and are formed through biogeochemical or mechanical processes [11]. Examples include clay minerals, volcanic ash, ocean spray, and naturally occurring iron and carbon nanoparticles (Figure 1) [10,11,12]. For example, volcanic ash contains a heterogeneous mixture of NPs, predominantly silica, aluminosilicates, iron oxides, titanium oxides, and trace metal-containing particles, whose composition varies depending on the volcanic source and eruption conditions [13]. On the other hand, ocean spray aerosols contain NPs composed primarily of sea salt, organic matter, sulfates, and biogenic compounds, with their composition influenced by seawater chemistry, biological activity, and atmospheric conditions [14].

In addition to geological origins, a significant subset of NONPs is generated by biological entities. A prominent example is the natural occurrence of biological magnetic nanomaterials, such as the biogenic magnetite Fe_3_O_4_ nanoparticles synthesized by magnetotactic bacteria. These biologically sourced nanoparticles exhibit exceptional crystallinity, narrow size distribution, and natural lipid membrane coatings, making them highly advantageous for advanced biomedical applications, including magnetic resonance imaging (MRI) and targeted drug delivery [15]. It is important to distinguish these inherently natural biological nanoparticles from those produced via sustainable nanoparticle synthesis, often referred to as “green synthesis”, which is an engineered, eco-friendly approach that employs biological agents such as plant extracts, fungi, or isolated microbial enzymes as reducing and capping agents to manufacture diverse nanoparticles without the use of toxic chemicals. While sustainable green synthesis provides a highly scalable, versatile, and environmentally safe approach to creating engineered nanomaterials, naturally formed biological nanoparticles like magnetosomes are synthesized in vivo under strict genetic control, offering unparalleled innate biocompatibility and precise structural characteristics that remain challenging to replicate artificially [16].

### 2.2. Incidental (Anthropogenic) Nanoparticles

**Figure 2:**
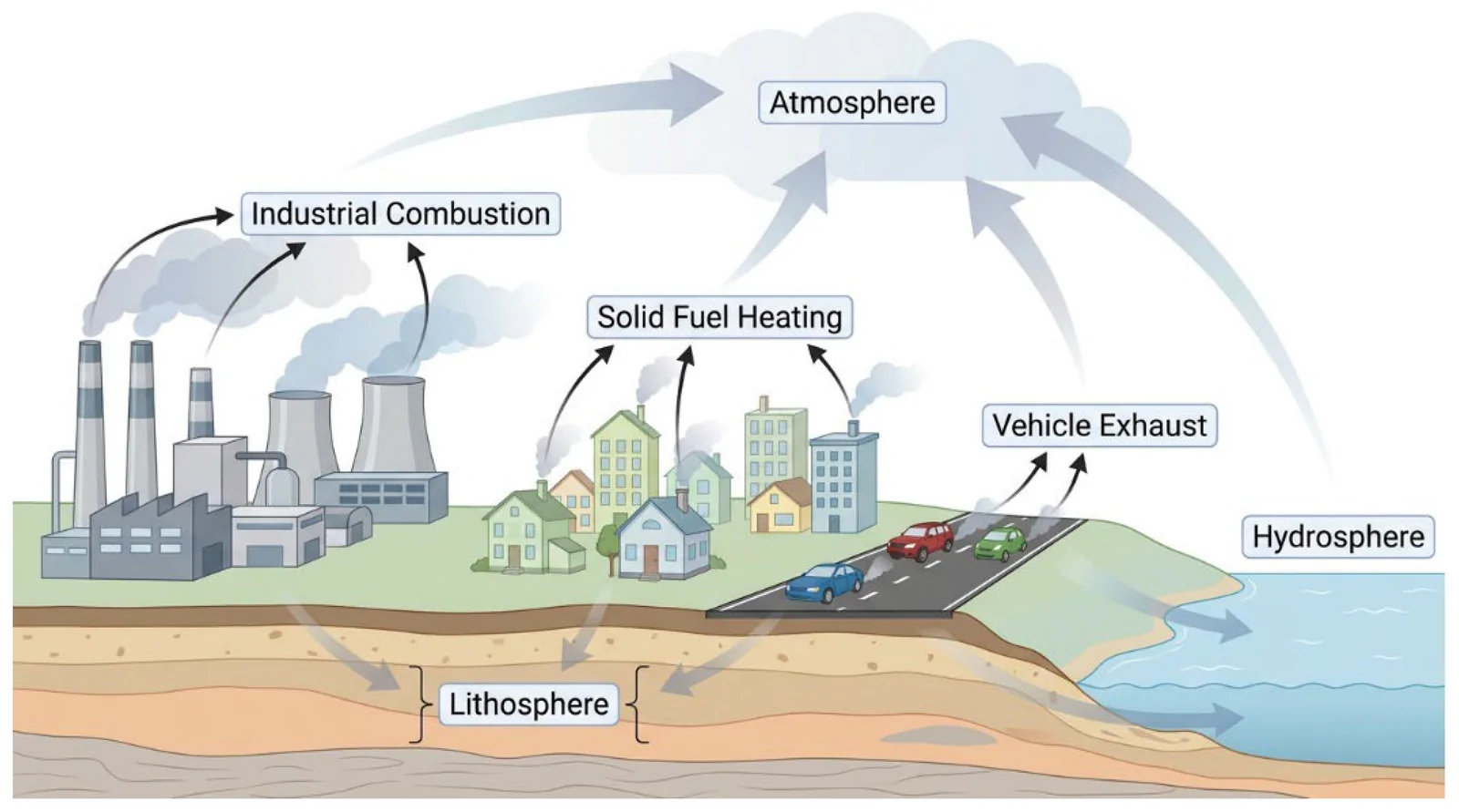
Origins and environmental pathways of incidental (anthropogenic) nanoparticles [10,11].

These NPs are unintentionally produced as by-products of human activities, such as vehicle exhaust, solid fuel heating, and industrial combustion [10,11]. Because they are emitted continuously and possess poorly controlled physicochemical characteristics, incidental nanoparticles constitute an important and often overlooked source of environmental exposure and potential adverse health effects. Both natural and incidental nanoparticles contribute heavily to exposure pathways in the atmosphere, hydrosphere, and lithosphere (Figure 2) [10,11].

### 2.3. Engineered Nanomaterials

**Figure 3:**
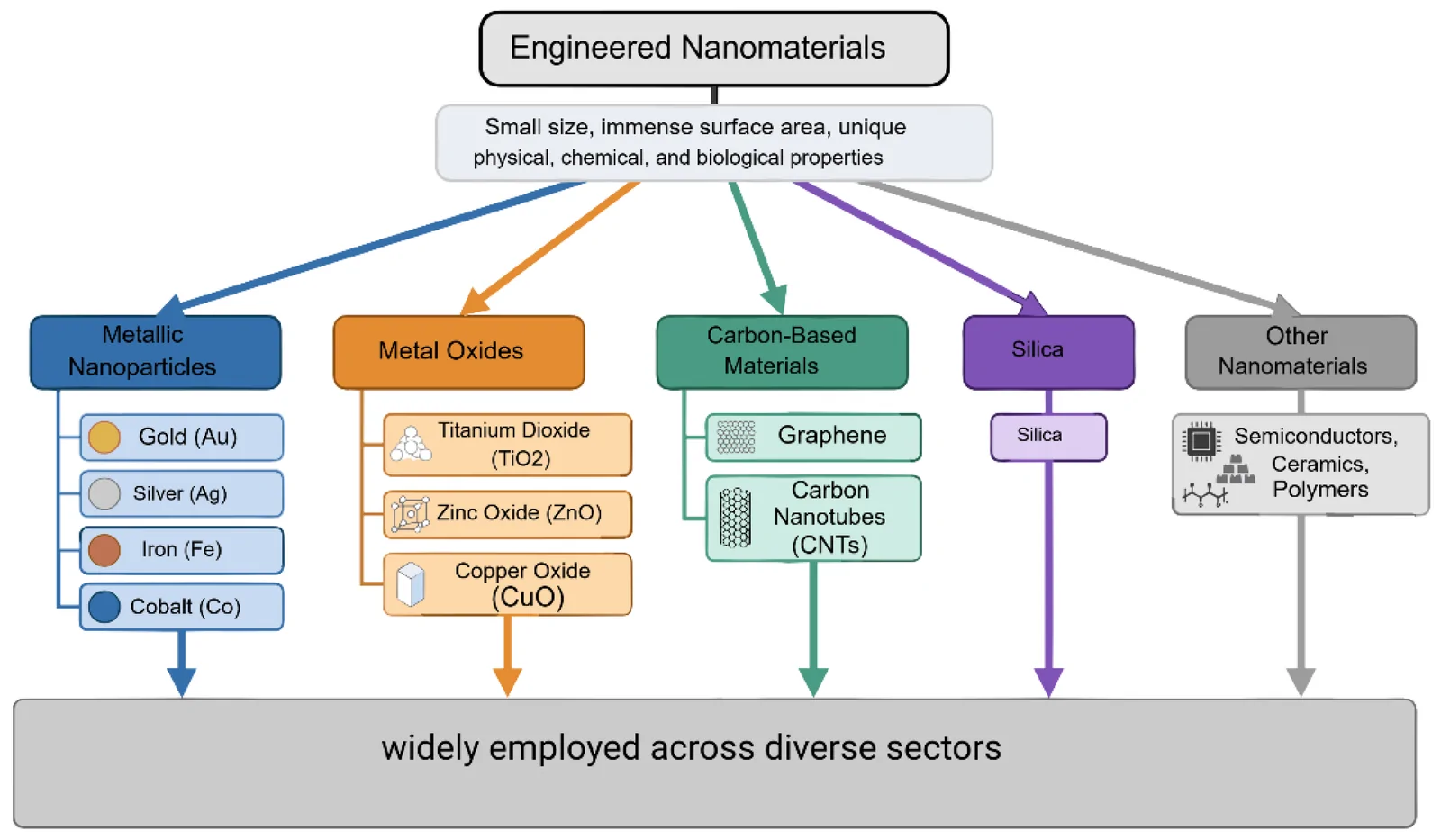
Classification and key properties of engineered nanomaterials [4,9,17].

Nanomaterials, as engineered structures manufactured with defined dimensions, encompass a broad and diverse range of classifications [4]. This range includes metallic nanoparticles (such as gold, silver, iron, and cobalt), metal oxides (like titanium dioxide, zinc oxide, and copper oxide), carbon-based materials (including graphene and carbon nanotubes) and silica, and other nanomaterials [4,9,17]. A wide variety of methods have been developed for their synthesis, and these approaches have undergone continuous refinement to enable precise control over particle size, shape, composition, crystallinity, and surface chemistry. Such advances have improved the reproducibility, scalability, and functionality of nanoparticles, allowing their physicochemical properties to be tailored for specific biomedical, industrial, and environmental applications [10]. In recent years, considerable attention has also been directed toward green and sustainable synthesis strategies as safer and often more efficient alternatives to conventional methods, yielding nanoparticles with reduced cytotoxicity and improved biocompatibility [18]. The unique physicochemical properties of engineered nanoparticles have enabled their widespread adoption in diverse technological and scientific domains (Figure 3) [4].

**Figure 4:**
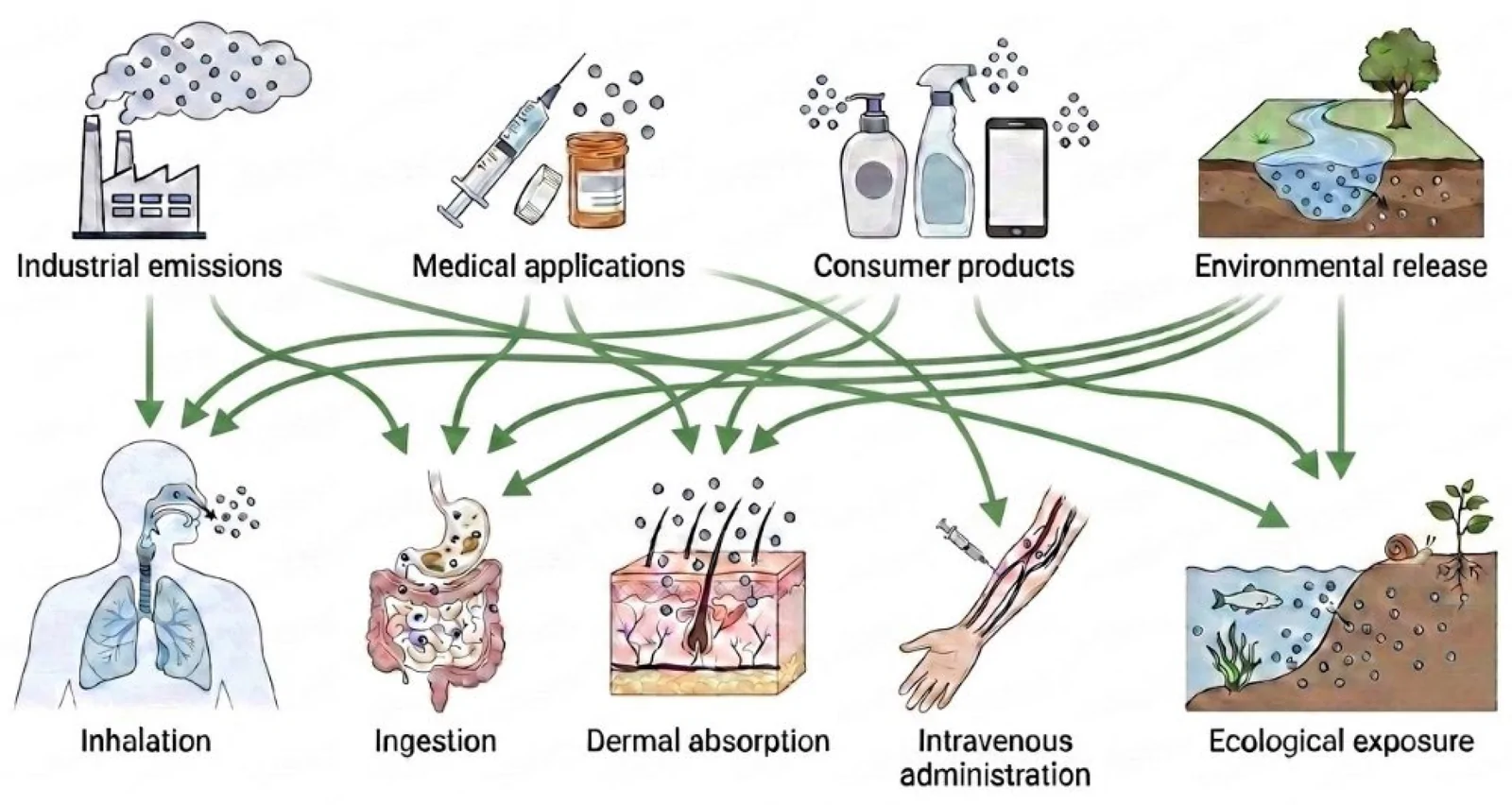
Major environmental and biomedical exposure pathways of engineered nanomaterials leading to potential human and ecological exposure [3,4,6].

Industrially, NPs are widely utilized in electronics, consumer cosmetics, paints, agriculture, food packaging, and environmental remediation technologies such as wastewater treatment [3,4]. In the biomedical field, their impact is revolutionary; they are employed for targeted drug delivery, gene therapy and medical imaging like MRI contrast agents, and advanced theragnostics, which combine both diagnosis and treatment into a single functional platform [3,4,9,17]. However, the widespread use of nanomaterials and the exponential growth in their production have created a significant gap between the rapid development of novel nanoscale formulations and the capacity to comprehensively evaluate their safety [4,17]. As the pace of nanomaterial innovation and commercialization continues to accelerate, experimental assessment of nanoparticle toxicity remains a major challenge owing to its complexity, time requirements, and high cost. Consequently, the rate at which new nanomaterials are introduced into research and commercial applications far exceeds the capacity to generate robust toxicological data, raising concerns regarding their safe and responsible implementation (Figure 4) [6].

## 3. Mechanisms of Nanotoxicity

The toxicity of nanomaterials is highly dependent on their physical and chemical properties. A primary parameter is particle size [4]. A smaller size significantly increases the surface area-to-volume ratio, enhancing the particle’s reactivity, cellular uptake, and ability to penetrate biological barriers [4,5]. Particle shape also influences toxicity [3,4]. Spherical particles exhibit different cellular internalization compared to elongated or fibrous shapes [5]. Furthermore, surface charge regulates toxicity [19]. Because cell membranes are negatively charged, cationic nanoparticles show stronger electrostatic interactions, leading to increased cellular uptake and cytotoxicity [4,5].

**Figure 5:**
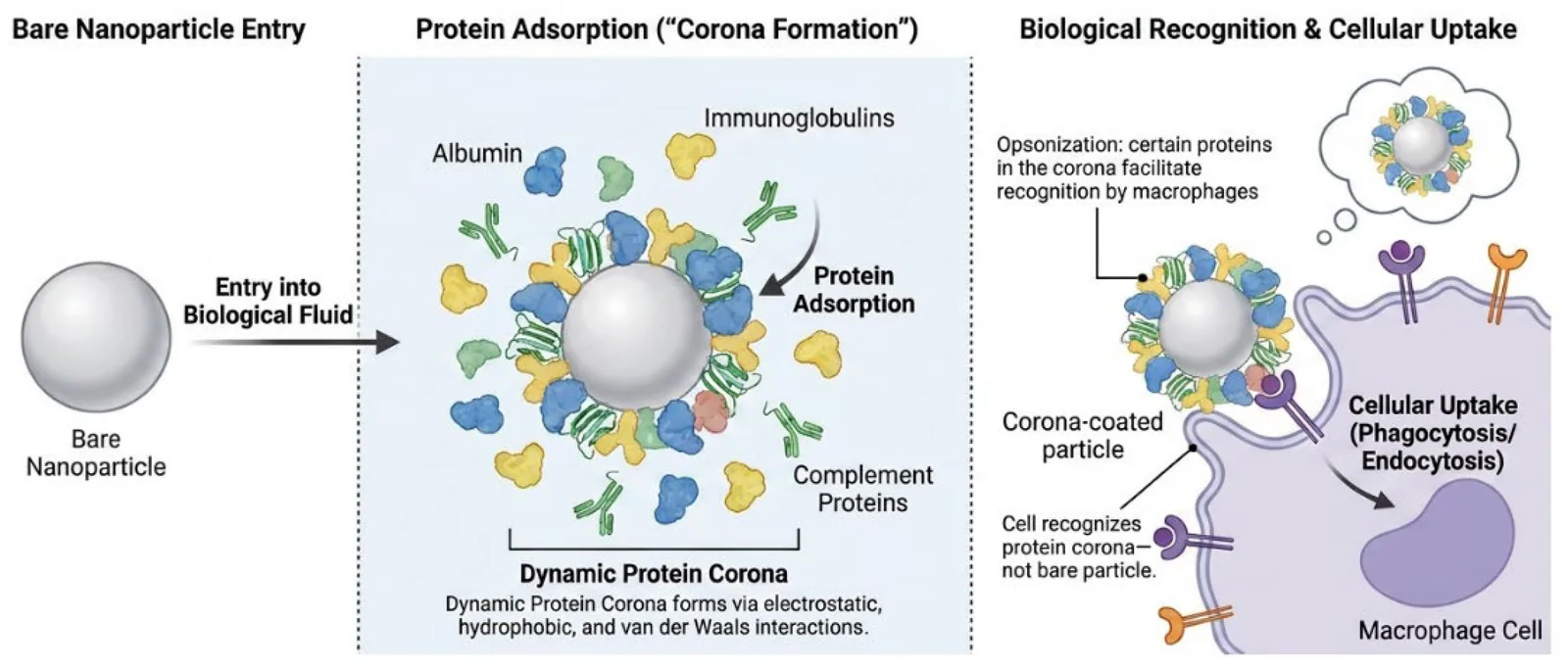
Formation of protein corona around nanoparticles alters their biological identity, influencing toxicity and biodistribution [3,4,19].

When nanoparticles enter biological fluids, proteins rapidly adsorb onto their surfaces to form a protein corona layer (Figure 5) [3,4,19]. Consequently, cells, including phagocytes, primarily recognize and interact with the protein corona rather than the bare nanoparticle surface, and the corona may either shield the nanoparticle from detection or facilitate its recognition by specific cellular receptors [19]. Interestingly, this shielding effect can sometimes be beneficial, as it protects healthy cells from the immediate, destructive damage that would otherwise be induced by bare, highly reactive cationic nanoparticles [3].

Furthermore, the specific composition of this corona critically determines the nanoparticle’s cellular uptake pathways, such as clathrin-mediated endocytosis, or phagocytosis [7,17]. In particular, the adsorption of certain serum proteins, such as non-specific immunoglobulins and complement factors, causes opsonization that act as highly visible molecular signals that alert the body’s immune system, specifically directing macrophages and the mononuclear phagocyte system to engulf and clear the nanoparticles from the biological system [20].

**Figure 6:**
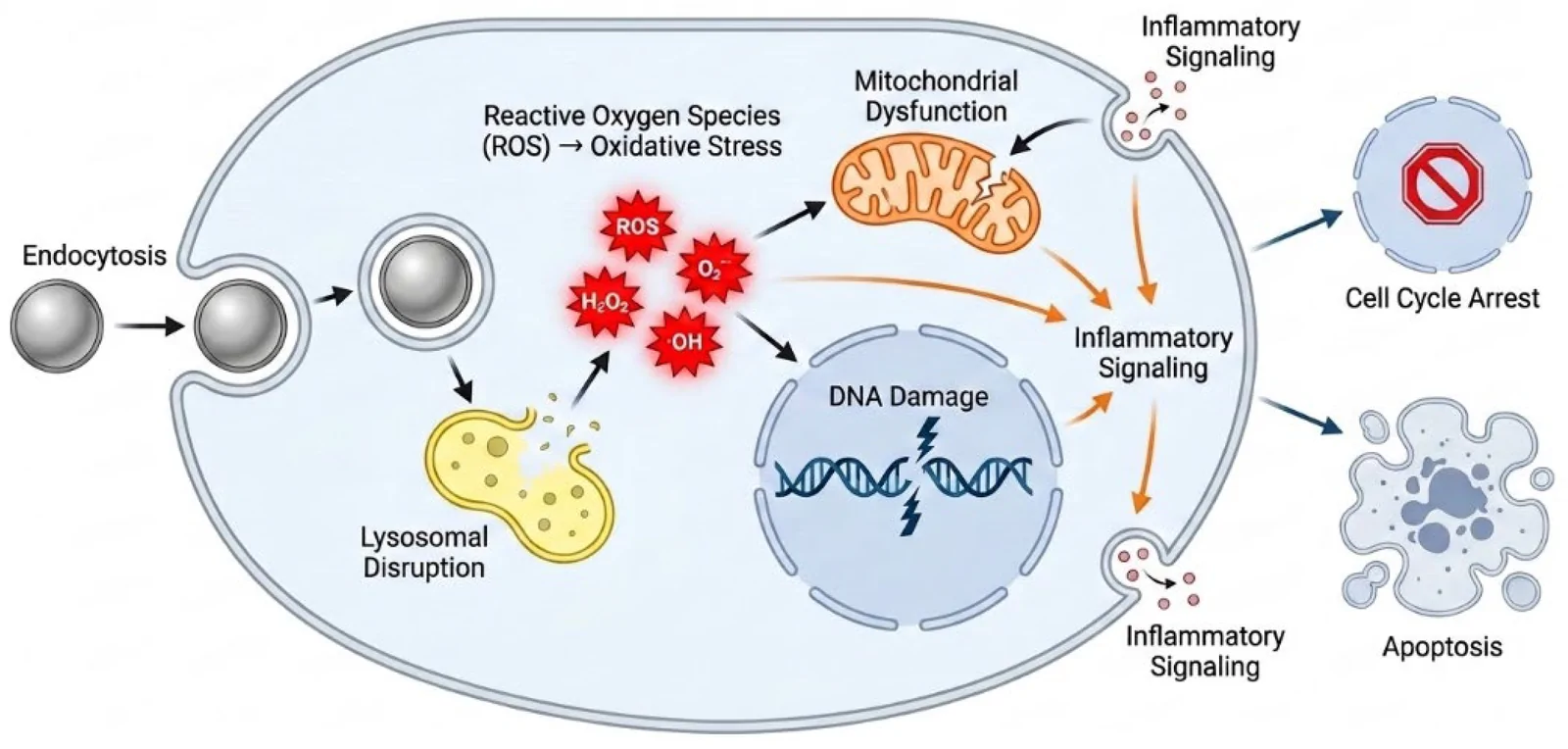
Cellular mechanisms involved in nanotoxicity including oxidative stress, inflammation, and genetic damage induced by nanoparticle interaction [3,4,6].

Following cellular internalization, nanoparticles induce cytotoxicity primarily through the excessive generation of reactive oxygen species (ROS), making oxidative stress one of the principal mechanisms underlying nanomaterial toxicity [3,4,6]. ROS production can occur through multiple pathways, including electron leakage from the mitochondrial electron transport chain, surface-mediated redox reactions, the release of redox-active metal ions, activation of inflammatory cells, and the intrinsic catalytic properties of certain nanoparticles, which enable direct electron transfer reactions with biological molecules [3,4,6,21,22]. Their large specific surface area, high surface reactivity, and tunable electronic properties can further promote the formation of superoxide anions, hydrogen peroxide, and highly reactive hydroxyl radicals. Simultaneously, excessive ROS production overwhelms cellular antioxidant defense systems. The resulting oxidative stress causes lipid peroxidation, protein oxidation, and DNA damage, and disrupts membrane integrity and mitochondrial functions that can lead to the activation of redox-sensitive signaling pathways involved in inflammation and stress responses. Persistent oxidative stress and mitochondrial dysfunction further amplify ROS generation, creating a self-perpetuating cycle that promotes genomic instability, DNA mutations, cell cycle arrest, and ultimately cell death through apoptosis, necrosis, or other forms of regulated cell death. These molecular and cellular disturbances contribute to the development and progression of numerous pathological conditions associated with nanoparticle exposure (Figure 6) [3,4,6,21,22].

To quantitatively evaluate the severity of NPs cytotoxicity, the half-maximal inhibitory concentration IC_50_ (half-maximal inhibitory concentration) serves as a critical benchmark, demonstrating that cellular susceptibility varies significantly depending on nanoparticle composition, surface capping, target cell line, and exposure duration. For example, recent evaluations of commercial colloidal silver formulations on human hepatoma (Huh-7) cell lines revealed high cytotoxic potency, yielding 24 h IC_50_ values as low as 0.62 µg/mL [23]. Similarly, biogenically synthesized silver nanoparticles produced via *Pulsatilla koreana* root extract exhibited dose-dependent cytotoxicity and ROS-driven apoptosis, demonstrating pronounced sensitivity against A549 human lung carcinoma cells line with an IC_50_ of 16.75 µg/mL [24]. The increased sensitivity of cancer cells to NPs may be attributed to their inherently elevated basal levels of ROS, rendering them more susceptible to further oxidative insults. Consequently, the additional pro-oxidant activity of nanoparticles exacerbates oxidative stress, ultimately promoting cancer cell death. Conversely, engineered nanoparticles often exhibit a wider therapeutic window and selective toxicity. In particular, the IC_50_ of plant-mediated selenium NPs tested against HepG2 liver cancer cell lines was 70.79 µg/mL, while exhibiting substantially lower toxicity toward normal human lung fibroblast cells (WI-38) with an IC_50_ of 165.5 µg/mL [25]. These quantitative metrics reinforce that even sub-micromolar or low physiological concentrations of specific engineered nanomaterials can trigger cell death [23,24,25].

Beyond localized cellular damage, the ability of nanoparticles that allows them to invade systemic circulation and bioaccumulate in vital organs leads to severe secondary toxicities [4,9]. In particular, excessive accumulation of nanoparticles in the liver and spleen, the primary organs responsible for sequestering circulating nanoparticles, can cause hepatic injury, macrophage hyperactivation, cytokine-mediated inflammation, and ultimately tissue necrosis [4,9].

Nanoparticles capable of crossing the highly selective blood–brain barrier accumulate in brain structures, such as the striatum. Here, they induce severe neurotoxicity through microglial activation and oxidative damage, and their persistent accumulation has been strongly linked to the development of neurodegenerative illnesses such as Alzheimer’s and Parkinson’s diseases [3,4,9]. Certain nanoparticles can penetrate the protective blood–testis and placental barriers, accumulating in reproductive tissues and disrupting sex hormone synthesis. They can also damage germ cells and disrupt normal spermatogenesis, potentially leading to infertility and transgenerational developmental toxicity [3].

While ultra-small nanoparticles, typically under 5.5 nm, are quickly cleared through the kidneys, larger or highly reactive NPs tend to accumulate in the proximal tubules, where they can induce nephrotoxicity, impair renal filtration, and cause tubular injury [4,26].

Additionally, through direct inhalation or redistribution via the bloodstream, nanoparticles can accumulate in respiratory tissues and exacerbate oxidative stress in alveolar epithelial cells, triggering widespread airway inflammation, alveolar wall thickening, and ultimately, pulmonary fibrosis [3,4].

## 4. Limitations of Current Testing, Solutions and Strategies

Historically, the initial screening of nanoparticle safety has depended heavily on standard two-dimensional (2D) in vitro cell cultures. These 2D systems are highly valued and universally utilized due to their high throughput, reproducibility, and cost-effectiveness [26,27].

However, 2D cell cultures force cells to grow on rigid, flat plastic surfaces, entirely stripping them of their natural three-dimensional architecture, extracellular matrix contacts, and complex cell-to-cell interplay [26,28]. Because they cannot mimic the dynamic microenvironment of native tissues, this leads to altered cellular behavior and the gradual loss of tissue-specific phenotypic and functional characteristics [26]. 3D models like organoids and microfluidic models are utilized to provide a more comprehensive understanding of nanoparticle transport and toxicity, and also restore spatial architecture and improve biological resistance to enhance physiological relevance [17,26,27,28]. On the other hand, advanced in vitro 3D models are complex to establish, as they are highly sensitive to temperature and humidity variations, and require optimized media; their accuracy can be hindered by particle aggregation or cell multilayering, and they are also more expensive [29].

Another layer of complexity arises from the significant discrepancy between the results obtained from conventional 2D in vitro cultures and complex in vivo models [20,27,30]. These differences make it challenging to accurately extrapolate toxicological data to humans [29].

The most glaring issue is the complete absence of fluid dynamics. In a living organism, blood and interstitial fluids are constantly moving. In a static 3D culture, however, the rate of diffusion is extremely low. For nanomedicine, this lack of flow causes rapid nanoparticle sedimentation [11]. Instead of dynamically circulating and penetrating tissues as they would in vivo, the nanoparticles simply settle out of the fluid [26]. This fundamentally alters transport kinetics and misrepresents true nanoparticle–cell interactions, often restricting the access of nanoparticles to the viable cells hidden deep within the center of a 3D structure [26,30]. Beyond transport mechanics, the static environment severely impacts cellular metabolism. As cells grow within a 3D construct, they rapidly consume the limited available oxygen and nutrients while simultaneously producing toxic waste products [26]. Because there is no continuous fluid flow to restore the system and carry away toxins, the culture medium must be replaced manually and periodically just to prevent the cells from dying [26]. Furthermore, this lack of flow makes it difficult to achieve a homogeneous distribution of extracellular matrix components throughout the construct, which is a significant drawback when attempting to faithfully mimic natural human tissue architectures [26]. Transitioning to 3D systems introduces major analytical hurdles. Traditional 2D cultures are grown on flat, transparent plastic surfaces, making them very easy to observe and analyze with almost any standard imaging technique [26]. In contrast, thick 3D culture constructs are much more difficult to image [26].

Ultimately, in vivo animal testing remains the most reliable method to evaluate the complex systemic interactions and dynamic biological functions within a whole organism that in vitro models cannot replicate [29]. While in vivo animal testing plays a crucial role in preclinical safety and efficacy assessment, substantial physiological differences between animals and humans often limit the translation of these findings to clinical outcomes [17,31]. However, the validation of in vitro–in vivo correlations through the integration of data from both testing approaches can significantly enhance the reliability of safety evaluations, leading to more accurate predictions of nanoparticle behavior and toxicity in vivo [20,32,33].

Nevertheless, achieving the above requires experimental conditions that closely mimic real-world exposure scenarios. NPs tend to aggregate or agglomerate in biological media, complicating toxicity testing by substantially and often unpredictably changing the behavior of these materials [4,5]. There is currently a lack of universally standardized characterization protocols [4,17]. In addition to these characterization challenges, traditional mass-based dose metrics are often inadequate for NPs. Determining realistic exposure requires evaluating surface area and particle number; thus, measuring precise, accurate exposure levels across a material’s life cycle remains difficult [4].

To overcome these barriers, toxicological evaluation is transitioning toward microfluidic technologies that create highly controlled, dynamic microenvironments [26]. Microfluidic platforms allow for the precise manipulation of fluid volumes, ensuring continuous nutrient supply, waste removal, and the maintenance of essential physiological parameters like pH, oxygen gradients, and fluid shear stress [26,28]. These micro-engineered devices integrate continuously perfused microchannels with living cells to faithfully simulate the fundamental functional units of human organs [26].

For example, organ-on-a-chip (OOC) systems are advanced microfluidic cell culture devices designed to simulate tissue and organs by forming a complex multicellular architecture, tissue-to-tissue interfaces, and dynamic biological microenvironments [26]. These models allow to track nanoparticle distribution and observe secondary toxicity, such as nanoparticle metabolism in an intestinal module, offering a testing environment that closely replicates complex human physiology [26].

To further elevate the predictive capability and analytical depth of these microphysiological systems, recent developments emphasize coupling organ-on-a-chip devices with integrated micro-transducers and advanced microscopy platforms [34,35]. By embedding miniaturized electrochemical, optical, resistive, and mechanical transducers directly within the microfluidic architecture, continuous, real-time, and multiparametric monitoring of critical microenvironmental parameters, including pH, oxygen gradients, glucose, lactate, or ROS, can be achieved without disrupting the ongoing cell culture [34]. Furthermore, coupling organ-on-a-chip devices with non-destructive optical readouts, such as label-free 3D quantitative phase imaging (QPI) and integrated fiber-optic microendoscopy, enables continuous visualization of cellular behavior, migration, and structural adaptations over extended exposure timelines without the perturbations associated with destructive cell staining [35]. This synergistic integration of in situ biosensing and high throughput imaging creates a powerful, non-invasive concept for dynamically evaluating nanotoxicity and cellular responses with exceptional physiological fidelity [34,35].

Because experimental biological assessments are demanding, slow, and expensive, they cannot keep pace with the exponential synthesis and market release of novel nanomaterials [6]. To resolve this situation, the field is rapidly adopting in silico methods, specifically through Quantitative Structure-Activity Relationship (QSAR) and nano-SAR modeling [6]. These computational models serve as high-throughput screening tools capable of predicting the potential toxicity of nanoparticles before they are physically synthesized [36]. They provide a rapid and cost-effective means of toxicity screening, reducing development costs and reliance on animal testing. Although not yet fully accepted by regulatory agencies, they represent a promising approach for future nanomaterial risk assessment [6,36].

**Figure 7:**
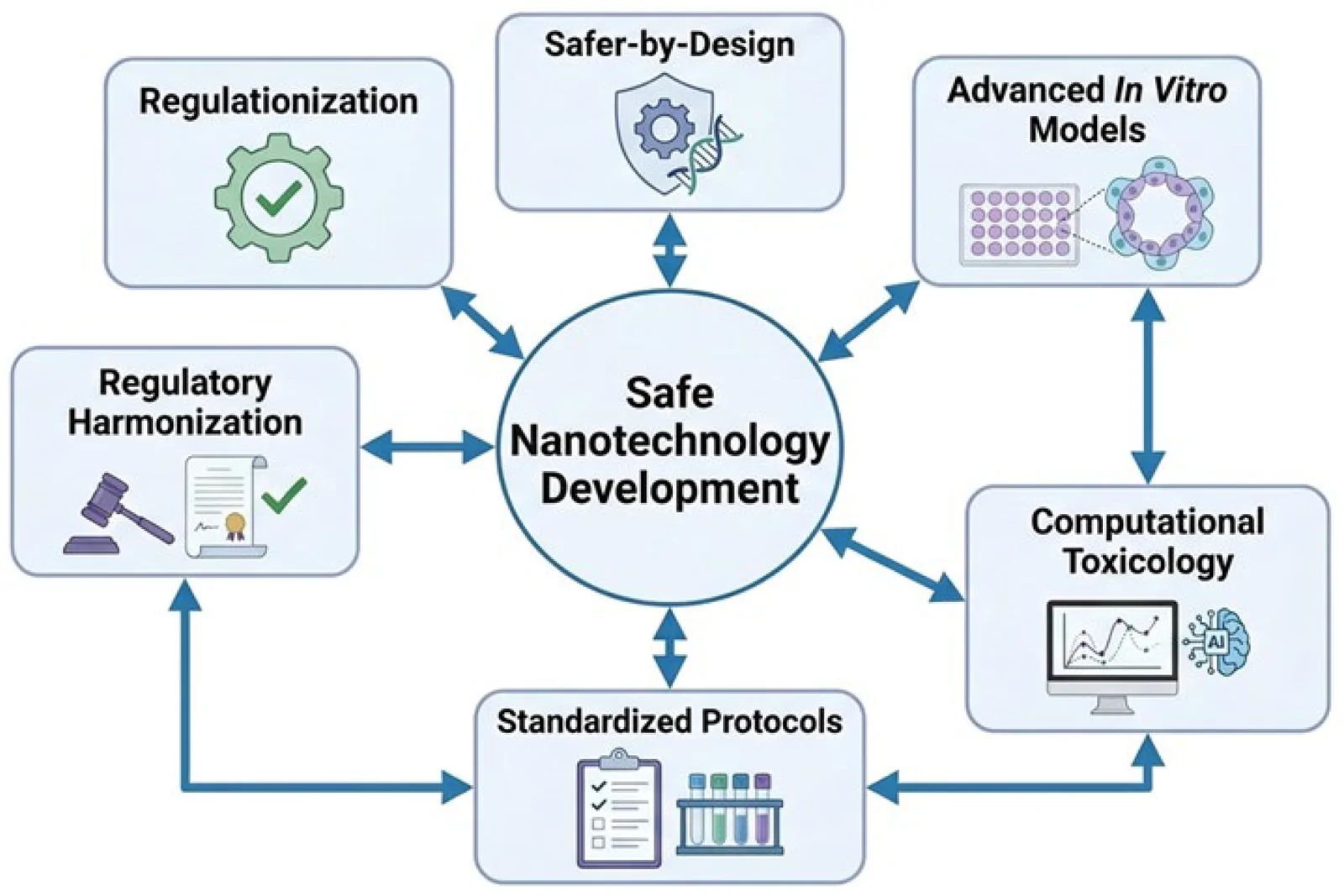
Integrated multidisciplinary strategies for overcoming nanotoxicology challenges [6,37].

Artificial intelligence (AI) and Machine Learning (ML) algorithms including random forests, k-nearest-neighbors, and support vector machines are trained on vast, heterogeneous datasets of toxicity measurements spanning diverse cell lines, environmental conditions, and organisms [6,8]. For metallic and metal-oxide nanoparticles, machine learning models have demonstrated that toxicity can be predicted with extreme accuracy using just two fundamental parameters: hydration enthalpy (HE) and the biological redox potential difference (Dbio) [6]. These advanced computational tools are not merely predictive but powerfully inductive by mapping untested nanoparticles to reveal the underlying mechanisms that drive toxicity, ensuring the creation of highly effective, inherently “safe-by-design” nanomedicines (Figure 7) [6,37].

## 5. Key Challenges in Nanotoxicology

Despite significant advancements in predictive modeling and testing platforms, nanotoxicology still faces profound challenges that impede the clinical translation and environmental risk assessment of nanomaterials, such as the “valley of death”, which is a phenomenon where many promising nanotechnology-formulated products fail to reach clinical approval due to the lack of harmonized definitions and the extreme difficulty in obtaining reliable evidence regarding their biological safety and in vivo behavior [38]. This challenge is further compounded by manufacturing impurities. For instance, contamination with bacterial endotoxins remains a grand challenge in immunological characterization, as it can severely confound toxicity data and obscure the true intrinsic hazard of the nanoparticle itself [20]. Additionally, at the computational and regulatory levels, the scientific community struggles with significant data curation challenges, including the absence of common languages, standardized reporting formats, and unified infrastructures needed to support reliable in silico modeling and decision-making frameworks [36].

**Figure 8:**
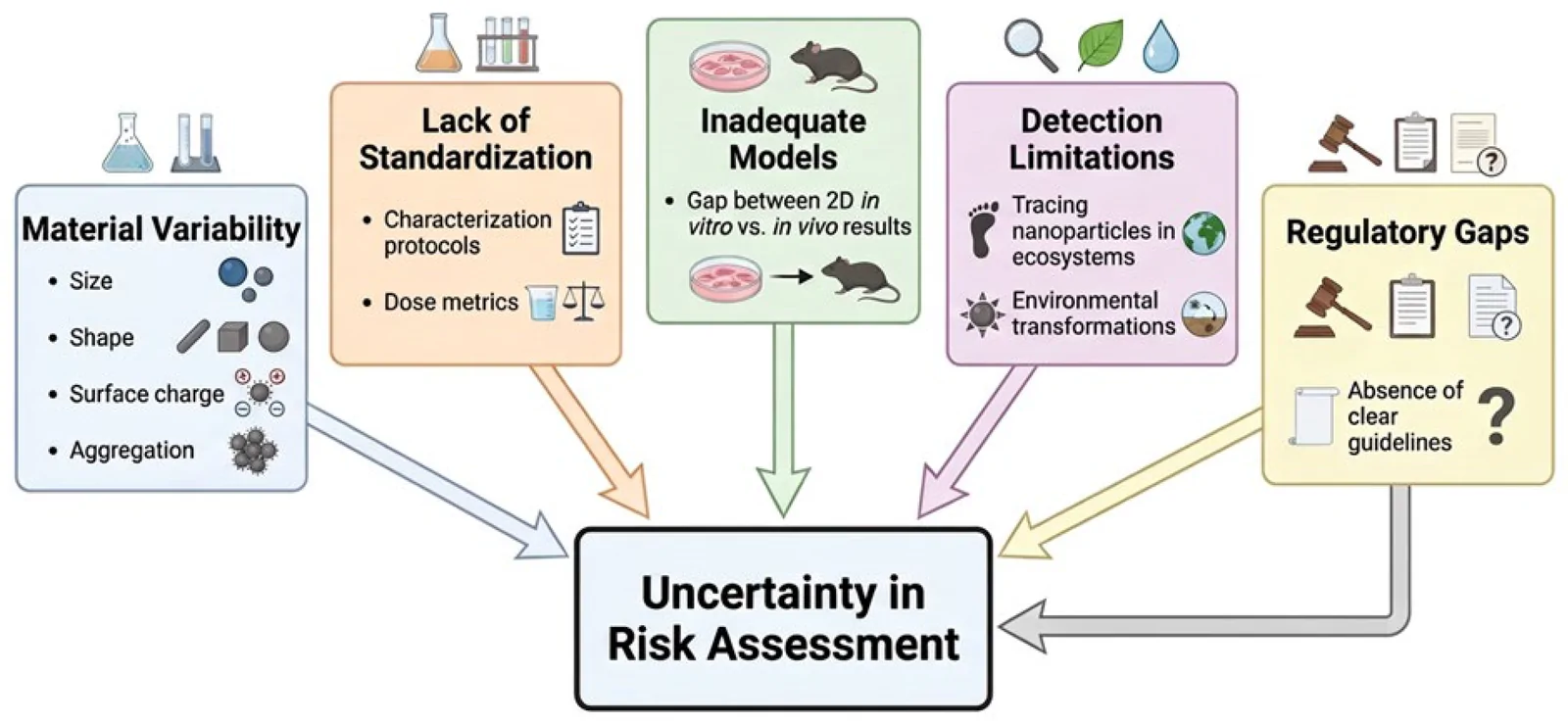
Major scientific and regulatory challenges limiting accurate nanotoxicological risk assessment [4,5].

Furthermore, tracing nanoparticles within the ecosystems is complex due to their environmental transformations, such as dissolution, sulfidation, and complexation [11,36]. Nanomaterials persist and bioaccumulate in soil ecosystems, impacting organisms across the food chain [4]. They can disrupt microbial communities, inhibit algal photosynthesis, and cause heavy metal accumulation, thereby making their effects on biological systems even more difficult to assess (Figure 8) [4,5].

## 6. Safer-by-Design: A Structural Approach to Mitigation

To address nanotoxicity proactively, the scientific community is shifting toward “safer-by-design” strategies [4,6]. This concept integrates safety into the earliest stages of nanomaterial development by optimizing physicochemical properties such as size, shape, surface chemistry, and composition to minimize hazards while maintaining therapeutic efficacy [4]. For example, the severe cytotoxicity often associated with strongly cationic surfaces which readily disrupt negatively charged cell membranes can be substantially decreased by neutralizing the surface charge or utilizing zwitterionic ligands to minimize non-specific biological binding [37].

In terms of composition, introducing specific dopants into the nanoparticle core can profoundly alter its dissolution rate and reactivity. A prominent example of this is the doping of highly reactive zinc oxide nanoparticles with iron, a structural modification that significantly decreases the rate of toxic metal ion dissolution and yields a much safer toxicological profile in vivo [5,23].

Additionally, to mitigate the significant toxicity associated with metal nanoparticles and the release of free metal ions, which can disrupt cellular homeostasis and induce excessive oxidative stress within cells, proper modification strategies are suggested [5]. Coating the external surface with polymers, such as polyethylene glycol (PEG), dextran, and poly (methyl methacrylate) (PMMA), provides steric stabilization that significantly reduces protein adsorption, hides nanoparticles from the immune system, and prevents drug release during systemic circulation, if conjugated [4,9,19].

Safer-by-design principles ensure that profound toxicity is precisely activated within tumor sites, helping to overcome cellular chemoresistance [22]. Validating these sophisticated mechanisms requires moving beyond standard testing to a predictive evaluation loop [4]. Computational in silico models, such as nano-Structure-Activity Relationships (nano-SAR), act as high-throughput screening tools to predict the toxicity of a formulation prior to its physical synthesis, relying on fundamental intrinsic properties like hydration enthalpy and conduction band energies [6,36]. These safe-by-design concepts can help evaluate following synthesis using microfluidic platforms, which simulate the complex 3D architecture [26]. By integrating predictive computational models with physiologically relevant microfluidic platforms, the dynamic transport and biological behavior of nanomaterials can be assessed more accurately, helping to ensure that next-generation nanomedicines are both effective and intrinsically safe before entering clinical trials [4,6].

Furthermore, the method of nanoparticle fabrication itself plays a critical role in safer-by-design strategies, making green synthesis a highly promising and sustainable alternative to conventional chemical manufacturing [18]. By utilizing biological platforms such as plant extracts, microorganisms, and algae, green synthesis eliminates the need for toxic reagents and significantly minimizes the generation of hazardous by-products [18]. Most importantly, nanoproducts obtained through these environmentally friendly methods are generally less toxic and exhibit improved biocompatibility compared to those produced using classical chemical methods [18]. This reduction in toxicity is largely because biologically mediated approaches naturally endow nanoparticles with biocompatible surface functional groups, avoiding the highly toxic synthetic chemicals and capping agents required in traditional synthesis [5]. For instance, comparative studies demonstrate that while chemically synthesized nickel nanoparticles induced a 24% mortality rate in human lymphocytes, their green-synthesized counterparts showed no observable toxicity at the exact same concentration [18]. Therefore, integrating green synthesis not only ensures ecological sustainability but fundamentally enhances the intrinsic safety of next-generation nanomaterials [18].

Beyond improving safety, the mechanisms of green synthesis enable one to customize the production of nanomaterials for specific, high-demand applications. During plant-mediated synthesis, phytochemicals such as polyphenols, flavonoids, and terpenoids function simultaneously as metal-reducing agents and natural stabilizers [18,39]. These plant-derived biomolecules rapidly adsorb onto the nanoparticle surface to form a dynamic layer that enhances colloidal stability, prevents harmful agglomeration, and directs favorable biological interactions [18].

Similarly, microorganism-mediated approaches influence the extracellular enzymes and metabolic processes of bacteria, fungi, and algae. Microalgae, in particular, are emerging as highly efficient “nano-factories” due to their innate capacity to hyperaccumulate heavy metal ions and perform bio-reduction under mild, ambient conditions [18].

Because of this enhanced biocompatibility and functionalization, the use of green-synthesized nanomaterials is rapidly expanding across biomedical, environmental, and agricultural sectors [18,39].

In nanomedicine, biogenic gold and silver and other nanoparticles are being extensively utilized for targeted drug delivery, photothermal cancer therapy, and advanced antimicrobial treatments, offering superior therapeutic indices compared to their chemically synthesized counterparts [18,39].

In environmental engineering, green-synthesized metal oxides (such as titanium dioxide and iron oxide) serve as highly effective photocatalysts and nano-biosensors capable of degrading toxic dyes, removing heavy metals, and monitoring wastewater pollution without introducing secondary hazardous chemicals [18,39]. Furthermore, in agriculture, these environmentally benign nanomaterials are being formulated into smart nanofertilizers and nanopesticides. These innovations improve targeted plant nutrient delivery and crop resilience while drastically reducing the toxic chemical load introduced into soil and aquatic ecosystems [18].

## 7. Assessment Framework

**Figure 9:**
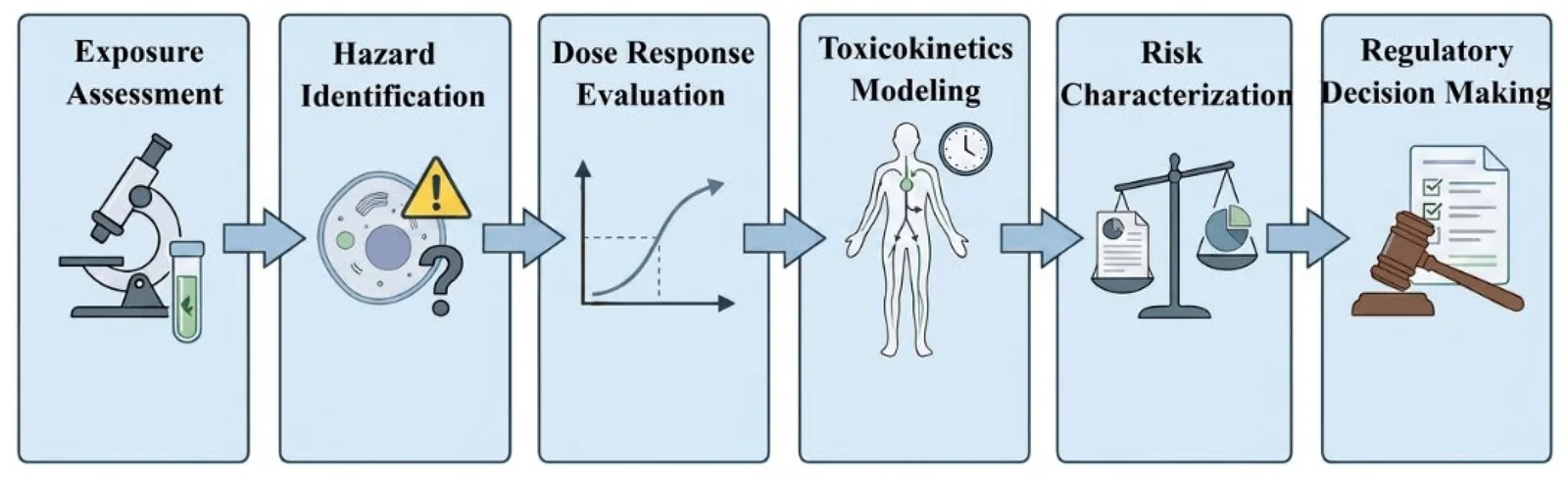
Standardized workflow for nanomaterial risk assessment integrating exposure and hazard data [4].

A comprehensive risk assessment framework for nanomaterials must integrate hazard identification, realistic exposure assessment across all life stages, and dose–response evaluation (Figure 9) [4].

The application of the traditional ADI concept to engineered NPs remains challenging because nanoparticle toxicity depends not only on dose but also on particle size, shape, surface area, surface chemistry, dissolution rate, agglomeration state, and protein corona formation. Consequently, many regulatory agencies recommend a case-by-case risk assessment, and for most engineered nanomaterials, no universally accepted ADI has yet been established [40].

Because biodegradable and dynamic nanomaterials are subject to spatial and temporal changes, risk assessments must utilize probabilistic approaches (e.g., Monte Carlo simulations and Bayesian methods) to account for changes in exposure and effects over time [41]. Effective risk characterization balances potential societal benefits against environmental and health risks [41].

## 8. Current Regulatory Status for Nanomaterial Safety Assessment and Related Issues

A number of initiatives address the search for unified testing standards to ensure the safe use of nanomaterials by developing universal and harmonized methodologies for evaluating their safety. Among them was a EU (European Union)-funded initiative, NANOMET, launched in 2020 to intensify and support the Organisation for Economic Co-operation and Development’s (OECD) work in developing tailored safety testing methods for nanomaterials [42]. The conclusion from these projects was that while many existing OECD Test Guidelines (TGs) for traditional chemicals are suitable for nanomaterials, specific test methods and guidance documents require adaptation to account for nanoscale behaviors.

The most important but also the best recognized limitation of existing regulatory guidance for toxicity assessment safety is that most toxicity guidelines were developed for conventional chemicals and bulk materials rather than engineered nanomaterials. There are several issues which have to be included in the safety testing framework, including specific physical and chemical parameters of nanoparticles, like particle size distribution, shape, surface area, and agglomeration state. Thus, testing adaptation should include data on how materials disperse and dissolve in different media to assess real exposure conditions. With respect to human health impact studies, it is important to note the limitations of the basic test systems used in toxicology studies, such as the Ames test for genotoxicity, which is based on the bacterial response [43]. Furthermore, MTT (3-(4,5-dimethylthiazol-2-yl)-2,5-diphenyltetrazolium) viability assay may give unreliable results [44]. Another critical aspect is the incorporation of assessment of protein corona formation, since this phenomenon substantially modulates the biological identity and bioactivity of nanoparticles [45]. This and other parameters, such as particle-number dosimetry or long-term accumulation assessment, are not yet fully standardized; however, some recommendations indicated in the next paragraph already exist. That makes nanomaterial safety assessment an active area of regulatory development. In this regard, it is important to note that conventional testing methods may need to be adapted, extended or, in some cases, replaced by equivalent tests specifically designed to ensure compatibility.

To ensure the safety and efficacy of nanomaterials, global regulatory bodies like the Food and Drug Administration (FDA) and European Medicines Agency (EMA) have established specialized laboratories, such as the US-NCL (United States Nanotechnology Characterization Laboratory) and the EU-NCL (European Union Nanotechnology Characterization Laboratory), which focus on standardizing analytical assays to meet rigorous regulatory standards [17]. These organizations actively promote the use of Standard Operating Procedures (SOPs) for physicochemical characterization, which helps to monitor batch-to-batch changes and ensures reliable data collection [17]. Furthermore, the OECD mandates that all non-clinical safety studies comply with Good Laboratory Practice (GLP) standards, ensuring that testing methods are uniform, robust, and capable of generating reproducible toxicological profiles [17].

OECD Nanomaterial Testing Guidance Documents (GDs) constitute an extensive framework of guidelines and guidance documents for the safety and regulatory evaluation of manufactured nanomaterials. It aligns nanomaterial testing with already existing safety frameworks to ensure global regulatory consistency and human and environmental safety. The framework includes several guidelines and guidance, with the Guidance on Sample Preparation and Dosimetry (GSPD), Test No. 125: Nanomaterial Particle Size and Size Distribution of Nanomaterials, and the Guidance Document for the Testing of Dissolution and Dispersion Stability. One of the key elaborations is GD No. 317, prepared for adapting standard aquatic and sediment toxicity tests to manufactured nanomaterials. It provides recommendations that take into account unique properties of nanoparticles, e.g., size, aggregation, dissolution, and surface chemistry, that can affect toxicity results. It focuses on proper nanoparticle characterization, dispersion preparation, exposure monitoring, test modifications, and data reporting to ensure reliable and reproducible hazard assessment of nanomaterials in aquatic environments [46]. Although GD 317 is an environmental ecotoxicology guideline, many of its principles, such as nanoparticle characterization, dispersion stability, dosimetry, and exposure assessment, are also fundamental to biomedical nanomaterial toxicity studies.

Another regulation, entitled Drug Products, Including Biological Products, that Contain Nanomaterials: Guidance for Industry, is an FDA guidance document that outlines considerations for the development, manufacturing, and evaluation of drug products containing nanomaterials [47,48]. It also emphasizes the importance of comprehensive physicochemical characterization, including particle size and its distribution, surface properties, and stability, as crucial for safety and efficacy assessment. Additionally, the guidance recommends estimation of how nanomaterials affect pharmaco-kinetics, biodistribution, immunogenicity, and toxicity compared with conventional formulations. It also highlights the need for appropriate quality controls and manufacturing processes to ensure product consistency throughout the product lifecycle. Overall, the document provides a risk-based framework to support the regulatory evaluation of nanomaterial-containing pharmaceutical and biological products.

There are also the EMA Reflection Papers on Nanomedicines, documents outlining the view of the Agency on specific issues concerning nanomaterials, including surface coatings: general issues for consideration regarding parenteral administration of coated nanomedicine products—Scientific guideline for Intravenous Iron-Based Colloids. The main considerations are included in the Reflection Paper on Nanotechnology-Based Medicinal Products for Human Use, which presents the basic requirements for the characterization of nanomaterials, emphasizing the importance of risk-benefit analysis [49].

Basically, the International Organization for Standardization ISO 10993 [50] Series (Biological Evaluation of Medical Devices) still remains the basic and the most widely used framework for evaluating the biological safety of medical materials, including nanoparticles. This guidance consists of several parts, such as ISO 10993-5 [51]: in vitro cyto-toxicity testing, ISO 10993-10 [52]: irritation and sensitization, ISO 10993-11 [53]: systemic toxicity, ISO 10993-3 [54]: genotoxicity, carcinogenicity, and reproductive toxicity, or ISO 10993-23 [55]: irritation testing.

At present, there is no single worldwide guideline that covers all necessary testing systems for medical nanomaterials and nanomedicines. Instead, regulatory agencies combine general biocompatibility standards, nanomaterial-specific guidance, and product-specific requirements. However, it is generally accepted that the combined application of ISO 10993 [50], the FDA Nanomaterial Guidance [47,48], OECD guidance documents for nanomaterial testing, and EMA reflection papers provides the foundation for the safety evaluation of nanomaterials.

In conclusion, despite significant advances in nanotoxicology, current regulatory guidelines still exhibit important limitations in the assessment of nanomaterial safety. Key challenges include the lack of standardized characterization methods, assay interference during cytotoxicity testing, limited guidance on long-term toxicity and immunotoxicity, and insufficient consideration of nanoparticle-specific properties such as protein corona formation, biodistribution, and accumulation in target organs. These gaps highlight the need for harmonized testing strategies and the development of nanomaterial-specific safety assessment frameworks for medical applications.

## 9. Future Perspectives

The future of nanotoxicology requires the integration of multi-omics approaches (genomics, transcriptomics, proteomics, and metabolomics) to build comprehensive Adverse Outcome Pathways (AOPs) like carbon nanotubes that map molecular initiating events to actual diseases [3,36]. Real-time environmental monitoring is becoming a reality through innovations like the IoT (Internet of Things)-integrated “NanoWatch” system and nano-biosensors, which track nanoparticle pollution in aquatic and atmospheric environments [56]. Furthermore, establishing global nano-safety databases (such as eNanoMapper) will facilitate data sharing [36]. Finally, the ethical considerations of nanotechnology must be addressed by ensuring environmental justice, protecting workers’ autonomy, and adhering to the principles of nonmaleficence [41,57].

## 10. Conclusions

As nanomaterials spread quickly, the application of nanomaterials necessitate a fundamental change in toxicological assessment and start of predicting safety risks before they occur [36]. Historically, the evaluation of nanoparticle toxicity has relied heavily on conventional in vitro cell cultures and in vivo animal models. But these methods are highly demanding, time consuming, and expensive, making them unable to keep pace with the exponential rate of nanoparticle innovation and time to market [6,30]. To overcome these limitations, the field must adopt proactive risk assessment driven by computer (in silico) modeling such as Quantitative Structure-Activity Relationship (nano-QSAR) models and machine learning algorithms that act as high-throughput screening mechanisms that can accurately predict a nanoparticle’s toxic class before it is physically synthesized [6,36]. These predictive models utilize fundamental physicochemical descriptors, such as the hydration enthalpy of released metal cations and the energy difference in the conduction band, to extract valuable information regarding how nanoparticles interact with biological systems [6].

Ultimately, shifting to predictive toxicology allows researchers to identify potential health risks early in development. This knowledge helps adjust a nanoparticle’s structure, guiding the creation of safer nanomedicines that maximize therapeutic benefit while minimizing harm [6,36].

## Data Availability

No new data were created or analyzed in this review article. Data availability is therefore not applicable.

## Abbreviations

The following abbreviations are used in this manuscript:

3D	Three-Dimensional
A549	Human Lung Carcinoma Cell Line
AgNPs	Silver Nanoparticles
AI	Artificial Intelligence
AOPs	Adverse Outcome Pathways
Dbio	Biological Redox Potential Difference
EMA	European Medicines Agency
EU	European Union
EU-NCL	European Union Nanotechnology Characterization Laboratory
FDA	Food and Drug Administration
GD/GDs	Guidance Document/Guidance Documents
GLP	Good Laboratory Practice
GSPD	Guidance on Sample Preparation and Dosimetry
HE	Hydration Enthalpy
HepG2	Human Liver Cancer Cell Line
Huh-7	Human Hepatoma 7 Cell Line
IC_50_	Half-Maximal Inhibitory Concentration
IoT	Internet of Things
ISO	International Organization for Standardization
ML	Machine Learning
MRI	Magnetic Resonance Imaging
MTT	3-(4,5-dimethylthiazol-2-yl)-2,5-diphenyltetrazolium
NANOMET	An EU-funded initiative supporting tailored safety testing methods for nanomaterials
nano-QSAR/QSAR	Quantitative Structure-Activity Relationship
nano-SAR	Nano-Structure-Activity Relationships
NMs	Nanomaterials
NONPs	Natural Nanoparticles
NPs	Engineered Nanoparticles
OECD	Organisation for Economic Co-operation and Development
OOC	Organ-on-a-Chip
PEG	Polyethylene glycol
Pk-AgNPs	*Pulsatilla koreana*-synthesized Silver Nanoparticles
QPI	Quantitative Phase Imaging
ROS	Reactive Oxygen Species
SeNPs	Selenium Nanoparticles
SOPs	Standard Operating Procedures
US-NCL	United States Nanotechnology Characterization Laboratory
WI-38	Normal Human Lung Fibroblast Cell Line
